# A FreeSurfer-compliant consistent manual segmentation of infant brains spanning the 0–2 year age range

**DOI:** 10.3389/fnhum.2015.00021

**Published:** 2015-02-18

**Authors:** Katyucia de Macedo Rodrigues, Emma Ben-Avi, Danielle D. Sliva, Myong-sun Choe, Marie Drottar, Ruopeng Wang, Bruce Fischl, Patricia E. Grant, Lilla Zöllei

**Affiliations:** ^1^Fetal-Neonatal Neuroimaging and Developmental Science Center, Boston Children's HospitalBoston, MA, USA; ^2^Laboratory of Computational Neuroimaging, AA Martinos Center, Massachusetts General HospitalCharlestown, MA, USA; ^3^Laboratories of Cognitive Neuroscience, Division of Developmental Medicine, Department of Medicine, Boston Children's HospitalBoston, MA, USA; ^4^Department of Electrical Engineering and Computer Science, Computer Science and Artificial Intelligence Laboratory, Massachusetts Institute of TechnologyCambridge, MA, USA

**Keywords:** MRI imaging, pediatrics, neuroimaging, segmentation, atlas

## Abstract

We present a detailed description of a set of FreeSurfer compatible segmentation guidelines tailored to infant MRI scans, and a unique data set of manually segmented acquisitions, with subjects nearly evenly distributed between 0 and 2 years of age. We believe that these segmentation guidelines and this dataset will have a wide range of potential uses in medicine and neuroscience.

## Introduction

Segmentations of volumetric magnetic resonance imaging (MRI) scans of the human brain have been widely used in neuroscience as an important basis for both structural and functional analysis. Segmentations can provide invaluable information on location, size, and shape of structures and their normal states, as well as on dynamic processes such as brain development and normal aging and disease progression. They may also serve as the basis for mapping activity for the purposes of functional imaging and connectivity studies.

Although many software packages have become available for automated or semi automated brain segmentation of the adult brain with good reliability and reproducibility (Cox, 1996; Dale et al., 1999; Fischl et al., 1999; Ashburner, 2012; Jenkinson et al., 2012), the same degree of development has not been achieved for the analysis of images of young children. This may be explained, in part, by the inherent lower quality of the images in terms of reduced resolution and more common motion artifacts, and by the relative lack of MRI intensity contrast differences between neighboring tissues, which is an important parameter when defining boundaries. Tissue contrast can be very subtle, almost indistinct and dynamically changing in a developing brain, with different boundaries becoming distinct at different ages. This is especially true during the first 2 years of life due to the myelin maturation process.

Many groups have explored methods for automatic or semi-automatic segmentation of infant brain MRI images (Prastawa et al., 2005; Nishida et al., 2006; Murgasova et al., 2007; Despotovic et al., 2010; Shi et al., 2010a,b, 2011; Yu et al., 2010; Wang et al., 2011; Gui et al., 2012; Choe et al., 2013), however, the primary focus has been the newborn stage (Prastawa et al., 2005; Nishida et al., 2006; Despotovic et al., 2010; Shi et al., 2010a,b, 2011; Yu et al., 2010; Wang et al., 2011; Gui et al., 2012), or other fixed ages (Murgasova et al., 2007; Choe et al., 2013; Dai et al., 2013), with fewer studies accommodating the full period of the first 2 years of life (Shi et al., 2011; Dai et al., 2013).

Although manually labeling regions of interest by an expert and summarizing such information in a training data set is still considered to be the most accurate way to establish brain atlases, to the best of our knowledge, there does not exist a fully manually labeled atlas for infants that is publicly available. This is probably due to the fact that the process of manual labeling is extremely time-consuming and tedious as well as often subjective necessitating intra- and inter-rater reliability estimates. Another concern is in regard to the number of structures delineated by the protocols of the automated or semi-automated segmentation algorithms, with the majority of them being limited to gray matter (GM), white matter (WM) and cerebral spinal fluid (CSF) (Prastawa et al., 2005; Shi et al., 2010a; Wang et al., 2011), and few providing more detailed brain parcellation (Murgasova et al., 2007; Yu et al., 2010; Shi et al., 2011; Gui et al., 2012; Choe et al., 2013).

**Figure 1:**
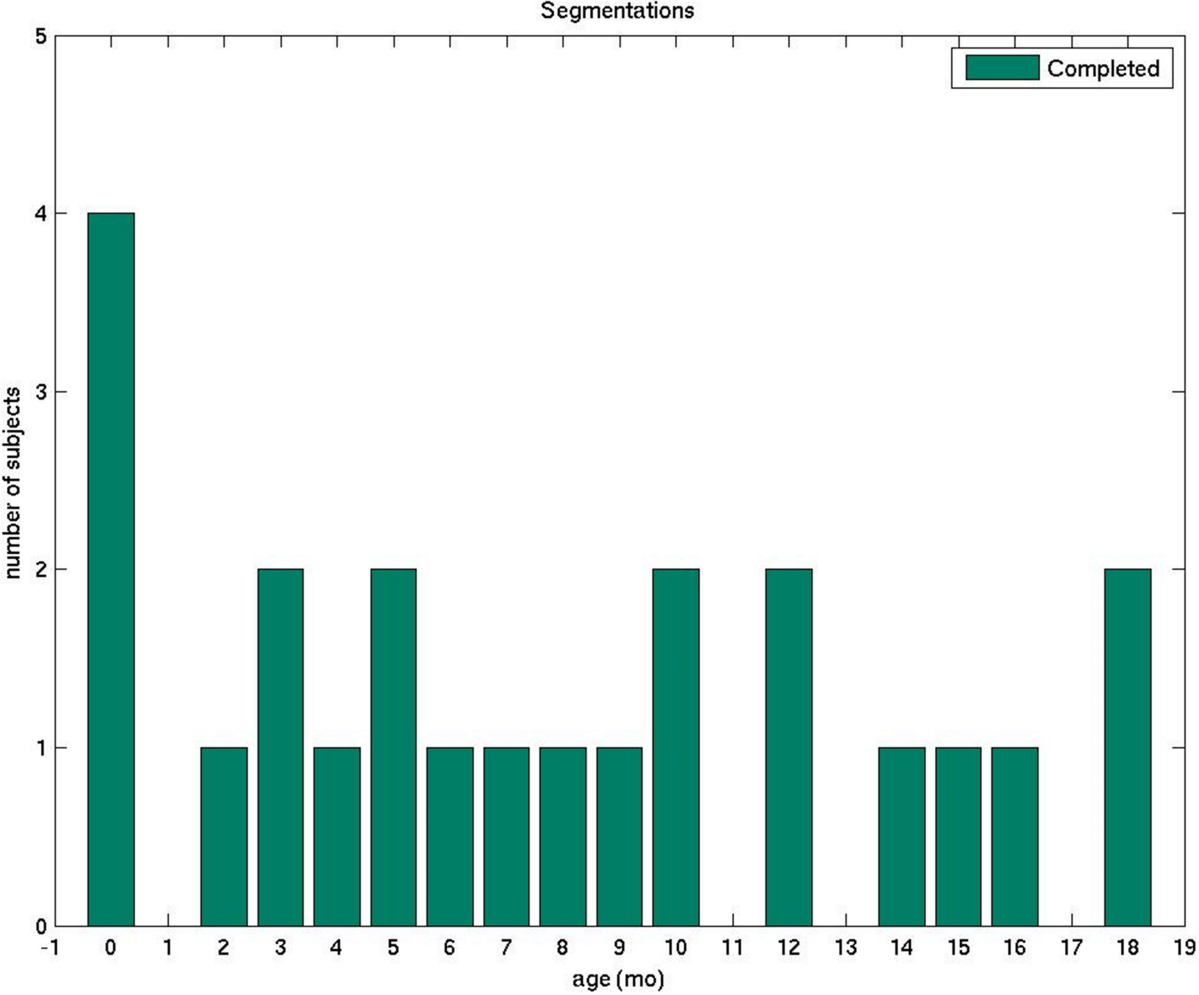
**The plot displays the number of manually segmented infant brain MRI scans by age**.

In this study, we describe a detailed protocol that we established and used for the manual segmentation of volumetric brain MRIs of infants whose ages span the first 2 years of life. We introduce a data set of 23 subjects that was processed using this protocol, with a relative uniform age distribution from the first day of life to 18 months (Figure 1, Table 1). With the exception of the cerebral and cerebellar cortex-white matter separation, which was performed only for neonates and subjects older than 11 months, all regions of interest (ROIs) were delineated in all subjects. We believe that the detailed description of our boundary decision-making process may potentially assist other groups studying similar populations. Furthermore, we are also planning on making an atlas generated from our training data sets available to the scientific community (as part of the FreeSurfer package), which can serve as a template in structural and functional studies and as a teaching tool for trainees. Since the set of segmented structures is similar to the FreeSurfer labels currently available for adults, this dataset and atlas will help facilitate comparisons between groups of different ages and the study of brain development beyond the second year of life. Nevertheless, our anatomical guidelines are general and valid for any image processing software.

**Table 1:** **Labels consistently segmented in our data set**.

Left/Right Thalamus	Left/Right Ventral Diencephalon
Left/Right Putamen	Mesencephalon
Left/Right Pallidum	Pons
Left/Right Caudate	Medulla
Left/Right Accumbens Area	Left/Right Cerebellum Cortex
Left/Right Hippocampus	Left/Right Cerebellum White Matter
Left/Right Amygdala	Vermis
Left/Right Lateral Ventricle	Left/Right Cerebral Cortex
3rd-Ventricle	Left/Right Cerebral White Matter
4th-Ventricle	

Our long-term goal is to create a reference data set for validation purposes and an atlas that will aid in the software development for fully automated brain segmentation tailored to the infant population. We aspire for such a software package to be as successful in its functionality and as widely used as FreeSurfer has been for adult brains (Fischl, 2012).

## Materials and methods

### Subject selection

We retrospectively selected brain images of 23 infants, ranging from newborns to 2-year-olds, scanned on one of two 3 Tesla magnets at Boston Children's Hospital (BCH) between 2009 and 2012. All the MRI studies were clinically indicated. To be included in the study, the subject's brain had to be considered structurally normal by both the attending pediatric neuroradiologist responsible for the exam as well as one of the pediatric neuroradiologists in our research team (PG or KMR). As a common event in the post delivery period, extracranial hematomas were not considered significant abnormality to exclude the subject.

The study was approved by the Committee on Clinical Investigation at BCH.

### Imaging acquisition

Scans were performed on a Magnetom Trio Tim 3 Tesla or on Magnetom Skyra 3 Tesla (Siemens Medical, Erlangen, Germany) with a 32-channel adult head coil. Multi-echo volumetric magnetization prepared rapid gradient echo (MPRAGE) sequences (van der Kouwe et al., 2008) with volume navigators (vNav) for motion correction (Tisdall et al., 2012) were obtained in the sagittal plane with an average image resolution of 1 mm isotropic, higher than the typical clinical infant acquisition standard. A full list of acquisition parameters is described in detail in Table 2. The subjects were imaged under sedation or during natural sleep. Images were assessed for quality and scans considered not suitable for segmentation, due to degradation by motion or other artifacts, were discarded.

**Table 2:** **Subject age (wks), gender (G) and MRI acquisition information: Geometry (FOV, %FOV, matrix, #slices, pixel res, slice thickness, orientation), Timing (TR, TE, TI, NEX, bandwidth), Excitation (X) (flip angle), Machine (field strength, manufacturer)**.

**Age**	**G**	**MRI Parameters**			
		**Geometry**	**Timing**	**X**	**Machine**
0.14	F	192; 100%; 0\192\192\0; 160; (1.00, 1.00); 1.00; PIL	2530 ms; (1.74, 3.60, 5.46, 7.32)ms; 1260.00 ms; 4; 651	7°	3T TimTrio
0.29	F	192; 100%; 0\192\192\0; 120; (1.00, 1.00); 1.00; PIL	2300 ms; (1.74, 3.60, 5.46, 7.32)ms; 1400.00 ms; 4; 651	7°	3T TimTrio
0.40	F	–; –; –\200\200\–; 176; (1.00, 1.00); 1.00; PIL	2530 ms; 3.39 ms; 1100.00 ms; 1; 195	7°	3T TimTrio
0.60	F	–; –; –\192\192\–; 176; (1.1458, 1.1458); 1.00; PIL	2530 ms; 1.66 ms; −1.00 ms; 4; 651	7°	3T TimTrio
10.60	F	–; –; –\192\192\–; 160; (1.00, 1.00); 1.00; PIL	2530 ms; 1.74 ms; −1.00 ms; 4; 651	7°	3T TimTrio
14.40	F	–; –; –\256\256\–; 144; (0.7812, 0.7812); 0.90; PIL	2000 ms; 2.58 ms; 900.00 ms; 1; 180	9°	3T Skyra
15.70	F	192; 100%; 0\192\192\0; 128; (1.00, 1.00); 1.00; PIL	2530 ms; (1.74, 3.60, 5.46, 7.32)ms; 1400.00 ms; 4; 651	7°	3T TimTrio
17.90	F	–; –; –\192\192\–; 160; (1.00, 1.00); 1.00; PIL	2530 ms; 1.74 ms; −1.00 ms; 4; 651	7°	3T TimTrio
21.00	F	192; 100%; 0\256\256\0; 160; (0.7656, 0.7656); 0.90; PIL	2000 ms; 2.59 ms; 900.00 ms; 1; 180	9°	3T Skyra
25.10	F	–; –; –\192\192\–; 128; (0.9375, 0.9375); 1.00; PIL	2530 ms; 2.24 ms; 1100.00 ms; 1; 199	7°	3T TimTrio
26.00	F	–; –; –\448\512\–; 176; (0.5, 0.5); 1.00; PIL	1950 ms; 2.26 ms; 900.00 ms; 1; 200	9°	3T TimTrio
33.90	M	–; –; –\220\220\–; 176; (1.00, 1.00); 1.00; PIL	2530 ms; 3.39 ms; 1100.00 ms; 1; 195	7°	3T TimTrio
35.40	F	–; –; –\192\192\–; 160; (1.00, 1.00); 1.00; PIL	2530 ms; 1.74 ms; −1.00 ms; 4; 651	7°	3T TimTrio
41.00	M	–; –; –\448\512\–; 352; (0.5, 0.5); 0.5; PIL	1950 ms; 2.26 ms; 900.00 ms; 1; 200	9°	3T TimTrio
45.30	M	–; –; –\192\192\–; 160; (1.00, 1.00); 1.00; PIL	2530 ms; 1.74 ms; −1.00 ms; 4; 651	7°	3T TimTrio
47.40	M	–; –; –\192\192\–; 160; (1.00, 1.00); 1.00; PIL	2530 ms; 1.74 ms; −1.00 ms; 4; 651	7°	3T TimTrio
55.40	F	–; –; –\192\192\–; 160; (1.00, 1.00); 1.00; PIL	2530 ms; 1.74 ms; −1.00 ms; 4; 651	7°	3T TimTrio
55.70	M	–; –; –\192\192\–; 160; (1.00, 1.00); 1.00; PIL	2530 ms; 1.74 ms; −1.00 ms; 4; 651	7°	3T TimTrio
63.30	M	–; –; –\192\192\–; 160; (1.00, 1.00); 1.00; PIL	2530 ms; 1.74 ms; −1.00 ms; 4; 651	7°	3T TimTrio
68.60	M	192; 100%; 0\192\192\0; 160; (1.00, 1.00); 1.00; PIL	2530 ms; (1.74, 3.60, 5.46, 7.32)ms; 1400.00 ms; 4; 651	7°	3T TimTrio
73.30	F	–; –; –\192\192\–; 176; (1.1458, 1.1458); 1.00; PIL	2530 ms; 1.66 ms; −1.00 ms; 4; 651	7°	3T TimTrio
80.00	F	–; –; –\256\256\–; 192; (0.8594, 0.8594); 0.90; PIL	2000 ms; 2.52 ms; 900.00 ms; 1; 180	9°	3T Skyra
80.10	M	–; –; –\448\512\–; 288; (0.5, 0.5); 0.5; PIL	1950 ms; 2.26 ms; 900.00 ms; 1; 200	9°	3T TimTrio

Missing information is indicated with “–.”

### MRI pre-processing

Images were de-identified and tissue segmentation of DICOM images was carried out using FreeView^1^, the visualization and editing tool of the FreeSurfer package.

### Segmentation process

All segmentations were performed in the native space of each acquisition (without any transformation to a standard analysis space) either by an experienced neuroradiologist or a specifically trained research assistant. A total of six trained segmenters carried out the segmentation process. For the sake of maintaining accuracy and consistency, all subjects were reviewed, corrected and finalized by the same neuroradiologist (KMR) based on the protocol described below. Structures were mostly delineated in the plane corresponding to their respective long axes or where boundaries with neighboring structures could be more accurately visualized. The resulting segmentations were always revised in all three orthogonal planes for a tri-dimensional accuracy check. The list of ROIs that we segmented for all of our training data sets is indicated in Table 1. In this segmentation process, we often found it useful to refer to the anatomical and histological scans published in Griffiths (2010) as guidelines and to boundary descriptions discussed in Makris et al. (2004).

Below we describe in detail the segmentation boundaries and decisions that we use in order to characterize our set of ROIs (Table 1).

### Thalamus

**Figure 2:**
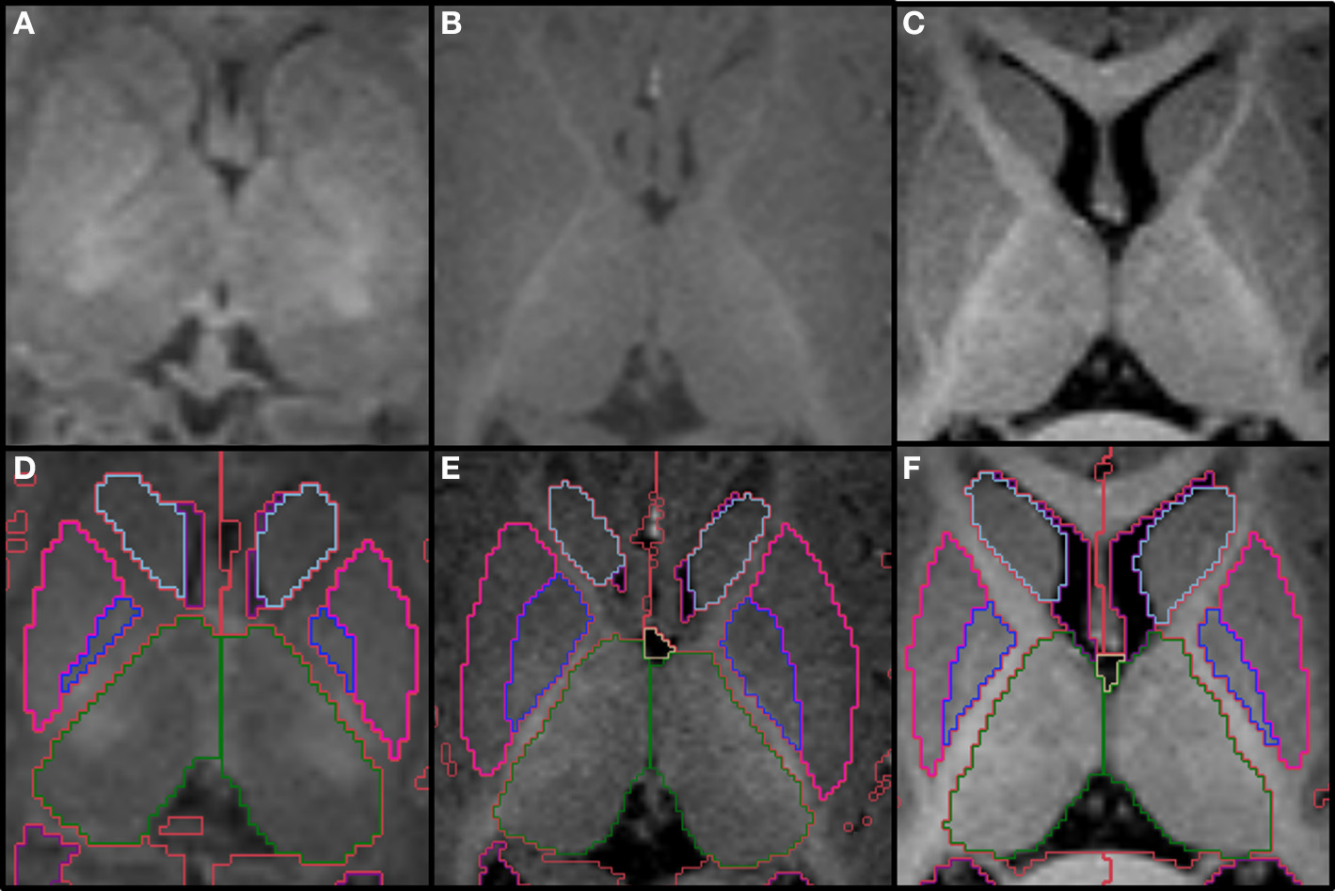
**Top row shows the myelination of the internal capsule in a neonate (A), 6 month old (B), and 12 month old (C)**. The bottom row **(D–F)** shows the pallidum (dark blue), putamen (pink), thalamus (dark green) and caudate (light blue), all segmented around the internal capsule. Other labels visible in the figure: lateral ventricles (purple), cerebral hemispheres in the neonate and 6 month-old (red), cerebral cortex in the 12 month-old (red), right cerebral white matter (bright green), left cerebral white matter (white). Note, on the boundaries of two structures the label colors mix.

The two thalami are usually the first structures to be segmented because they are the major central gray matter nuclei and they often serve as a reference for the segmentation of adjacent structures. We define the boundaries of the thalamus by the body of the lateral ventricles and the transverse fissure superiorly and medially, by the ventral diencephalon and mesencephalon inferiorly, by the third ventricle inferiorly and medially, and by the hippocampus and crus of the fornices posteriorly. The hypothalamic sulcus may help identify the inferior border in sagittal plane. In axial plane, the caudothalamic groove may be used as a reference for the anterior limit and the posterior limb of the internal capsule for the lateral limit, while in coronal plane, the caudothalamic groove becomes the supero-lateral limit of the thalamus with the posterior limb of the internal capsule as the lateral limit. In the neonatal brain, the internal capsule is only partially myelinated and therefore it appears thinner and with lower signal intensity. The T1 signal progressively increases as myelin matures, making segmentation in older infants easier (Figure 2). The two prominences on the posterior-lateral-inferior surface of the thalamus correspond to the geniculate bodies. They form the most inferior part of this structure, neighboring the perimesencephalic cistern and the choroid fissure. In the coronal and axial views, the geniculate bodies appear as if touching the hippocampus inferiorly and posteriorly, and the brainstem medially.

### Putamen and pallidum (lentiform nucleus)

**Figure 3:**
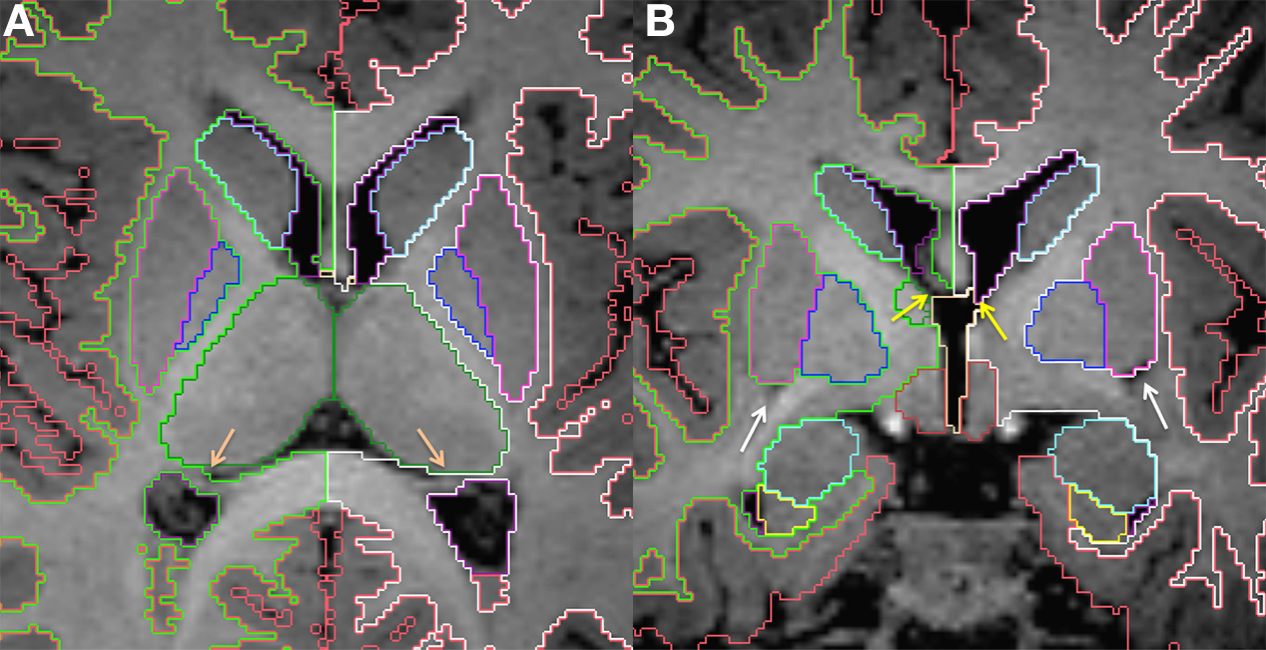
**Axial (A) and coronal (B) slices at the level of the basal ganglia in a 15 month old**. The putamen (pink) and pallidum (dark blue) segmented together roughly make a sideways triangle shape. The dark spots shown in (**B**, white arrows) correspond to the lateral lenticulostriate artery and helps identify the inferior border of the putamen. The lateral ventricles (purple) are also displayed. In **(A)**, the salmon colored arrows point to the fornices, which helps delineate the medial border of the lateral ventricles at the level of transverse fissure. In **(B)**, yellow arrows point to the foramen of Monro on each side. Other labels visible in the figure: caudate (light blue), thalamus (dark green), cerebral cortex (red), right cerebral white matter (bright green), left cerebral white matter (white), VDC (dark red), amygdala (celeste), hippocampus (yellow). Note, on the boundaries of two structures the label colors mix.

**Figure 4:**
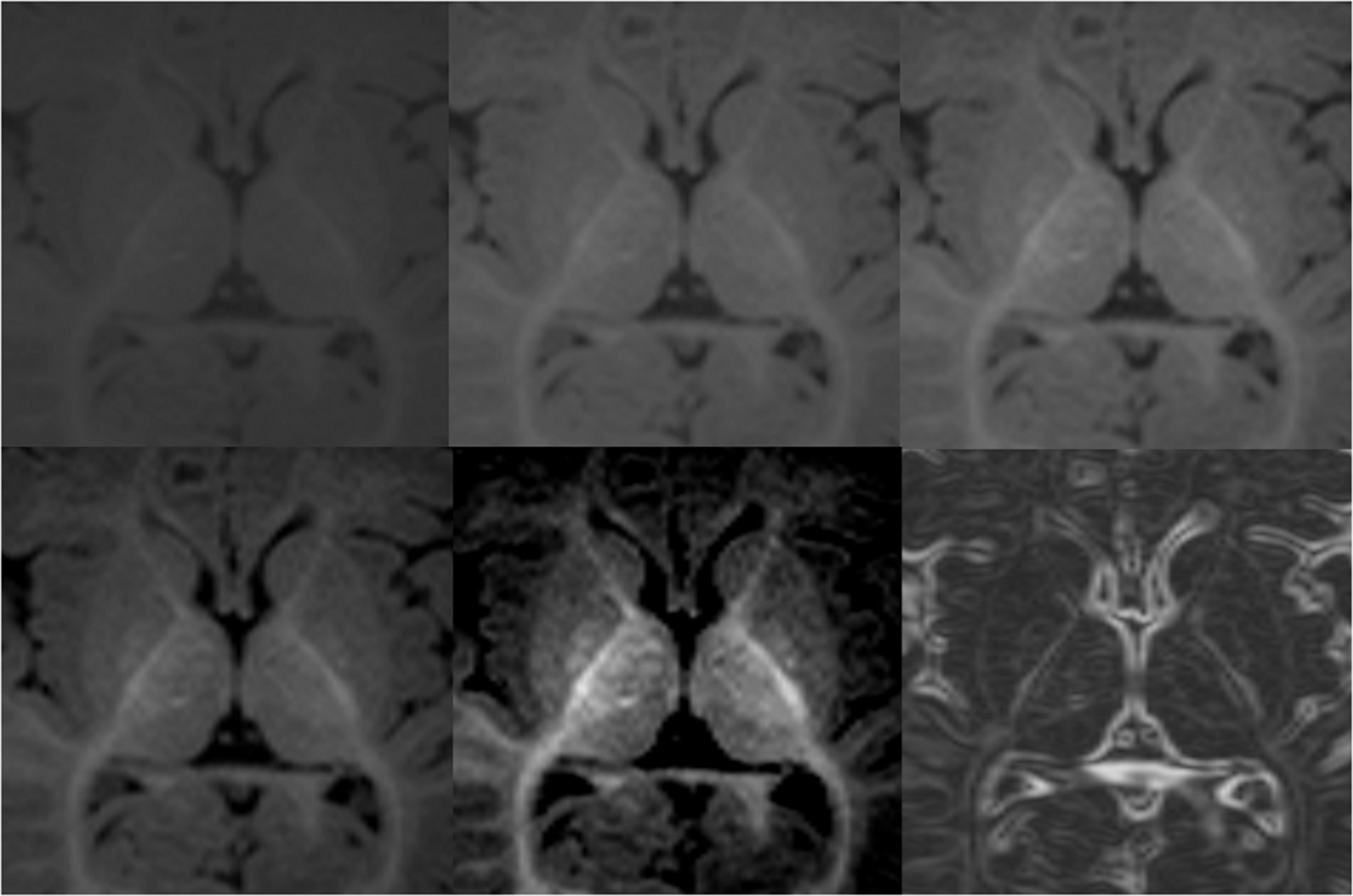
**Demonstration of variations in conspicuity of the borders of the lenticular nucleus with changes in the window-level settings in a 6-month-old**. The images with higher contrast allow for easier definition of boundaries. High contrast images should be used as guidance of shape and expected boundary, with final adjustments made using the average standard windows and width levels used for segmentation of the remainder of the brain structures. This correction is made to prevent false reduction in volume of high intensity structures, which may appear smaller on high contrast images. Bottom right picture shows results after Sobel filtering.

The putamen and pallidum (or globus pallidus) are deep gray matter nuclei grouped together in a lens shape, hence the name lentiform nucleus (Figure 3). They are located anterior and lateral to the thalamus, and posterior and lateral to the caudate, and are separated from these structures by the posterior and anterior limbs of the internal capsule, respectively. At the level of the expected inferio-lateral border of the lentiform nucleus, it is possible to identify one main T1 hypointense spot on coronal plane that corresponds to one lateral lenticulostriate artery. This is used as a reference for the inferior border of the lentiform nucleus in this plane. The external capsule defines the lateral border of the lentiform nucleus, which may be sometimes difficult to visualize when myelination has not yet occurred in this region. Prior knowledge of the anatomical shape and adjustment of the window-level settings in order to increase contrast differences are crucial when defining this boundary. This is demonstrated on Figure 4. Evaluation of the shape in the 3 planes also helps delineate the boundaries. Some segmenters have found the use of Sobel filters for edge detection to be helpful when lack of contrast differences are a challenge. Furthermore, it is also important to distinguish the globus pallidus from the putamen, where the former has slightly higher signal intensity. The subsegmentation of globus pallidus in medial and lateral is not performed due to insufficient contrast differences in this age group.

### Caudate

**Figure 5:**
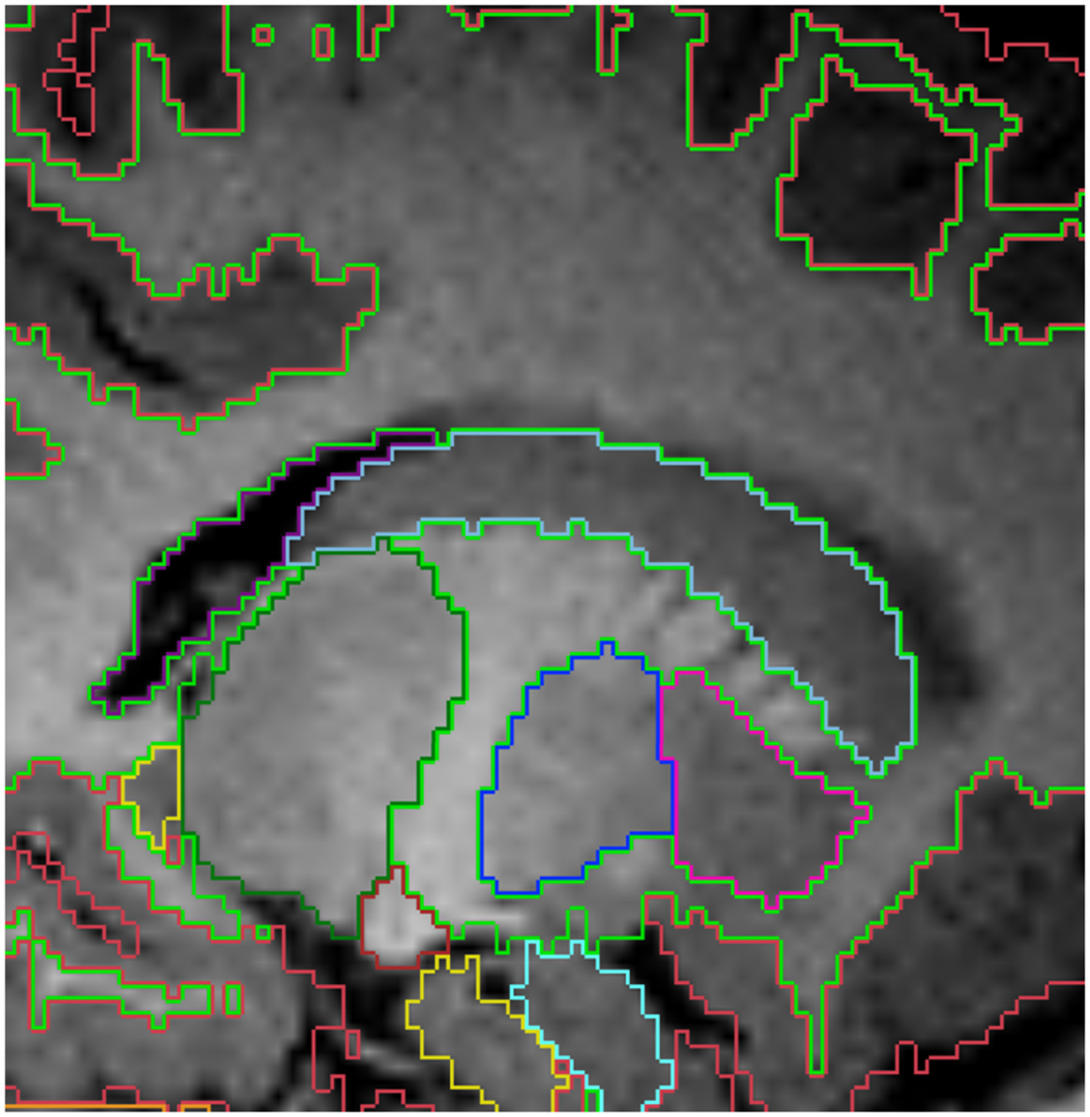
**Sagittal view of the segmented lateral ventricles (purple) and the caudate (light blue) in a 15 month old**. Other labels: cerebral cortex (red), right cerebral white matter (bright green), thalamus (dark green), putamen (pink) and pallidum (dark blue). Note, on the boundaries of two structures the label colors mix.

This pair of elongated, comma shaped gray matter structures run along the lateral margins of the lateral ventricles, which comprises most of its medial and superior borders (frontal horn) at the level of the caudate head, medial and superior borders (body of the lateral ventricle) at the level of the caudate body and medial inferior border (temporal horn) at the level of the caudate tail (Figure 5). The inferior and lateral border is formed by the internal capsule at the level of the caudate head, the corona radiata at the level of the caudate body and the posterior limb of the internal capsule/optic radiation at the level of the caudate tail. The inferior border of the caudate head neighbors the accumbens nucleus and the caudate tail touches the posterior aspect of the thalamus.

### Nucleus accumbens

**Figure 6:**
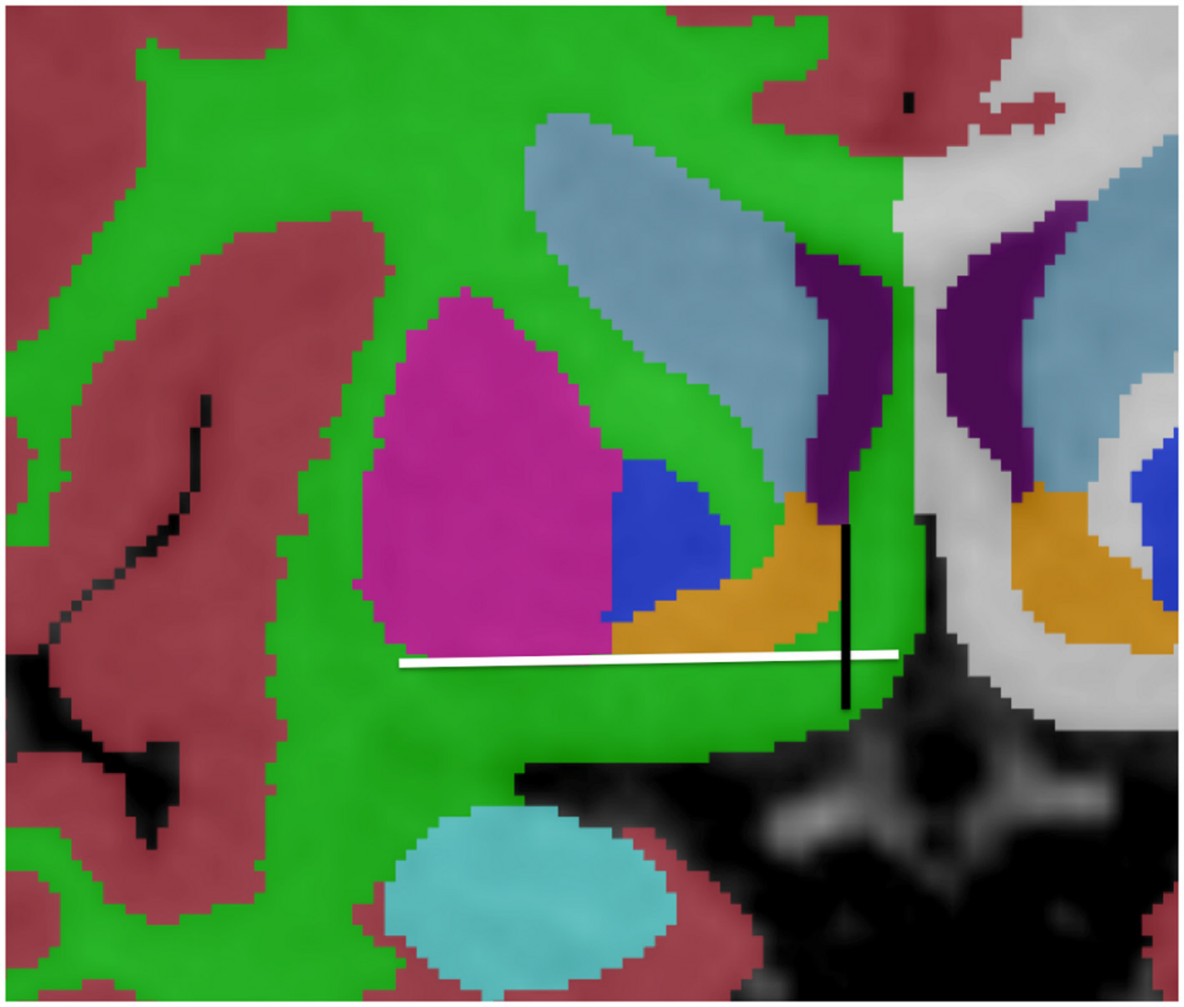
**Representation of the convention for accumbens (orange) delineation**. The white line drawn parallel to the orbital surface and the inferior border of the putamen (pink) helps defining the inferior border of the accumbens, while the perpendicular black line, running from the inferior tip of the lateral ventricle (purple) helps defining the lateral border. Other labels displayed: cortex (red), right cerebral white matter (bright green), left cerebral white matter (white), caudate (light blue), pallidum (dark blue) and amygdala (celeste).

The accumbens is a gray matter nucleus located inferiorly to the caudate head. It is so closely related to the putamen and caudate that the boundaries between these structures are not visible when using currently available MRI techniques. For this reason, a convention is necessary in order to trace this structure. We start delineating the accumbens in the coronal slice immediately after the first slice where we can see both the caudate and putamen. We continue delineating the accumbens in the following slices, from anterior to posterior. The coronal slice immediately anterior to the anterior commissure should be the most posterior slice with nucleus accumbens. When it is not possible to visualize the inferior border of the accumbens in an unmyelinated brain, we draw a line parallel to the orbital surface on the coronal plane, starting from the inferior tip of the putamen. This line connects to a perpendicular line that starts at the inferior tip of the lateral ventricle. This second line will be the medial limit of the accumbens. The medial-inferior border at the level of this intersection should be made round. The superior borders of the accumbens are the caudate medially, the lentiform nucleus laterally and the internal capsule centrally (Figure 6).

### Hippocampus

**Figure 7:**
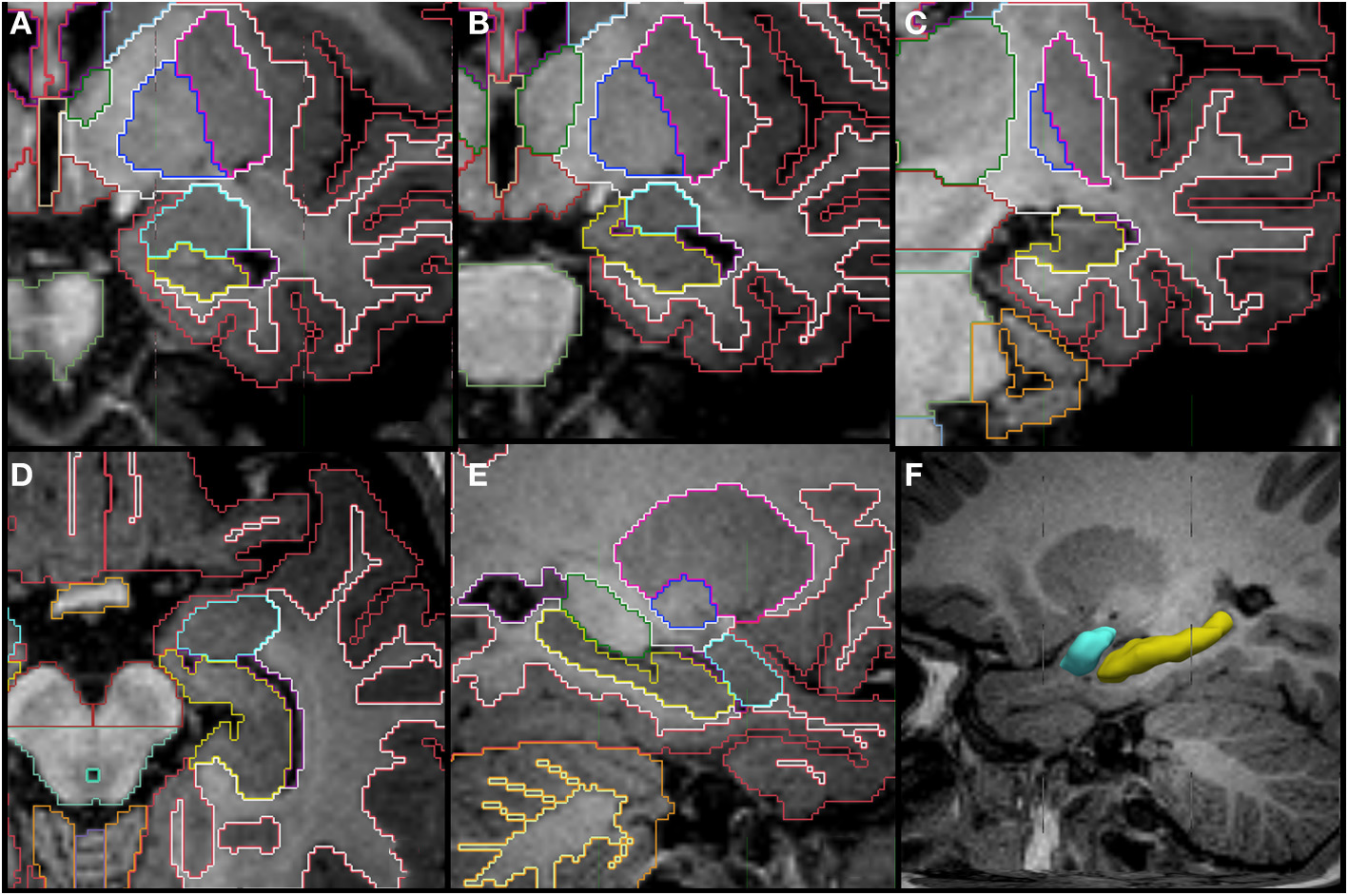
**Coronal (A–C), axial (D), sagittal slices (E,F) and a tridimensional representation of the segmentation of the hippocampus (yellow) and amygdala (celeste) in a 14 month old**. The amygdala appears superior to the hippocampus posteriorly and then as a round structure by itself in the more anterior portion. Other labels in the figures: cerebral cortex (red), cerebral white matter (bright green), thalamus (dark green), putamen (pink), pallidum (dark blue), cerebellar hemisphere (orange), cerebellar white matter (pale yellow in E).

The hippocampus is a key structure in the limbic system and corresponds to the elongated protuberance on the inferio-medial aspect of the temporal horns of the lateral ventricles (Figure 7). The lateral ventricle constitutes the superior and lateral borders of the hippocampus, while the inferior border is the white matter of the parahippocampal gyrus. Attention should be given to the change in contrast between hippocampus and white matter with age while delineating the inferior hippocampal border. The hippocampus is hyperintense relative to the white matter below during the neonatal period and becomes hypointense, as myelination in the temporal lobes occurs. In the medial superior aspect of the hippocampus there is a thin strip of white matter, the fimbria, which is labeled as hippocampus. The point where the fimbria separates from the hippocampus and becomes the crus fornicis, which is approximately at the level of the splenium of the corpus callosum, corresponds to the posterior border the hippocampus. The fornices are labeled as white matter.

### Amygdala

**Figure 8:**
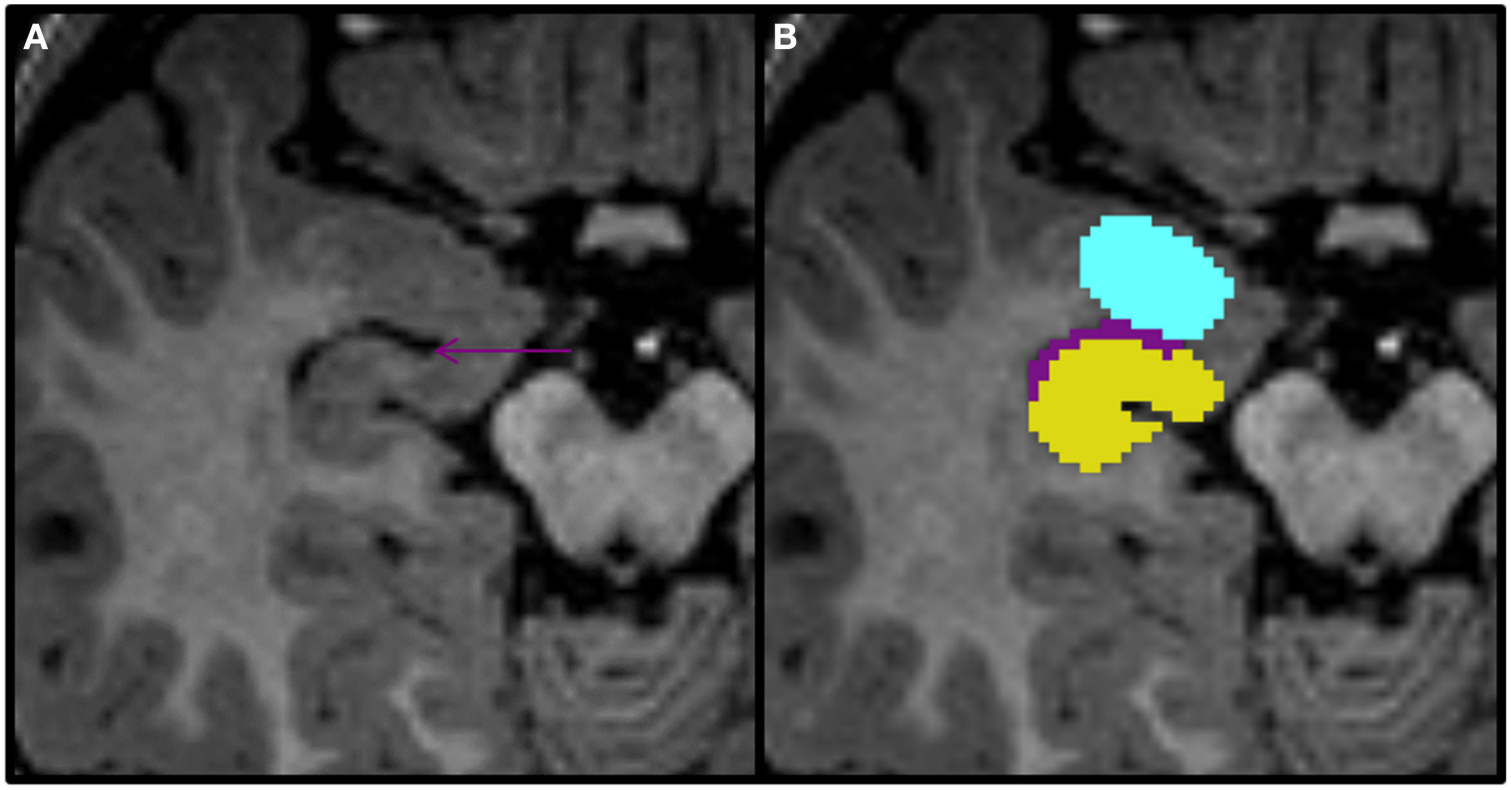
**Axial view of the amygdala (celeste), lateral ventricle (purple), and hippocampus (yellow) segmented in a 15-month-old**. In this view you can see that the uncal recess of the lateral ventricle (purple arrow) defines the border between the amydgala posteriorly and the hippocampus anteriorly.

The amygdala is also part of the limbic system and is closely related to the hippocampus. It is located in the medial temporal lobe in a position that is superior and anterior to the hippocampal head (Figure 7). It has a round shape in the coronal view and appears almond shaped in the sagittal view. Although, in the most anterior coronal slices, the inferior border of the amygdala is flattened and, combined with the hippocampus, make a round shape separated by a small pocket of CSF. This CSF pocket corresponds to the uncal recess of the temporal horn and it helps to identify the border between amygdala and hippocampus in the axial plane (Figure 8). The amygdala can be difficult to differentiate from the adjacent cortex due to similarity in signal intensity. The T1 contrast difference is more evident between the amygdala and the white matter that surrounds it laterally. It is often helpful to look at the shape of this structure in multiple planes.

### Lateral ventricles

The lateral ventricles correspond to the CSF filled cavities within each cerebral hemisphere and are relatively straightforward to segment. One particular area of possible difficulty is the anterior aspect, at the level of the interventricular foramina, where the lateral ventricles communicate with the third ventricle. At this location, the inferior border of the septum pellucidum can be used to define the inferior limit of the lateral ventricles in coronal plane. It can also be difficult to differentiate the lateral ventricles from the CSF in the medial aspect of the transverse fissure, particularly at the level of the occipital horns. In this case, the fornix should be used as a reference to define the medial border, with the lateral ventricle not extending medially to it on either side. Note that we do not have a separate label for the choroid plexus; it is included as part of the ventricles.

### Third ventricle

**Figure 9:**
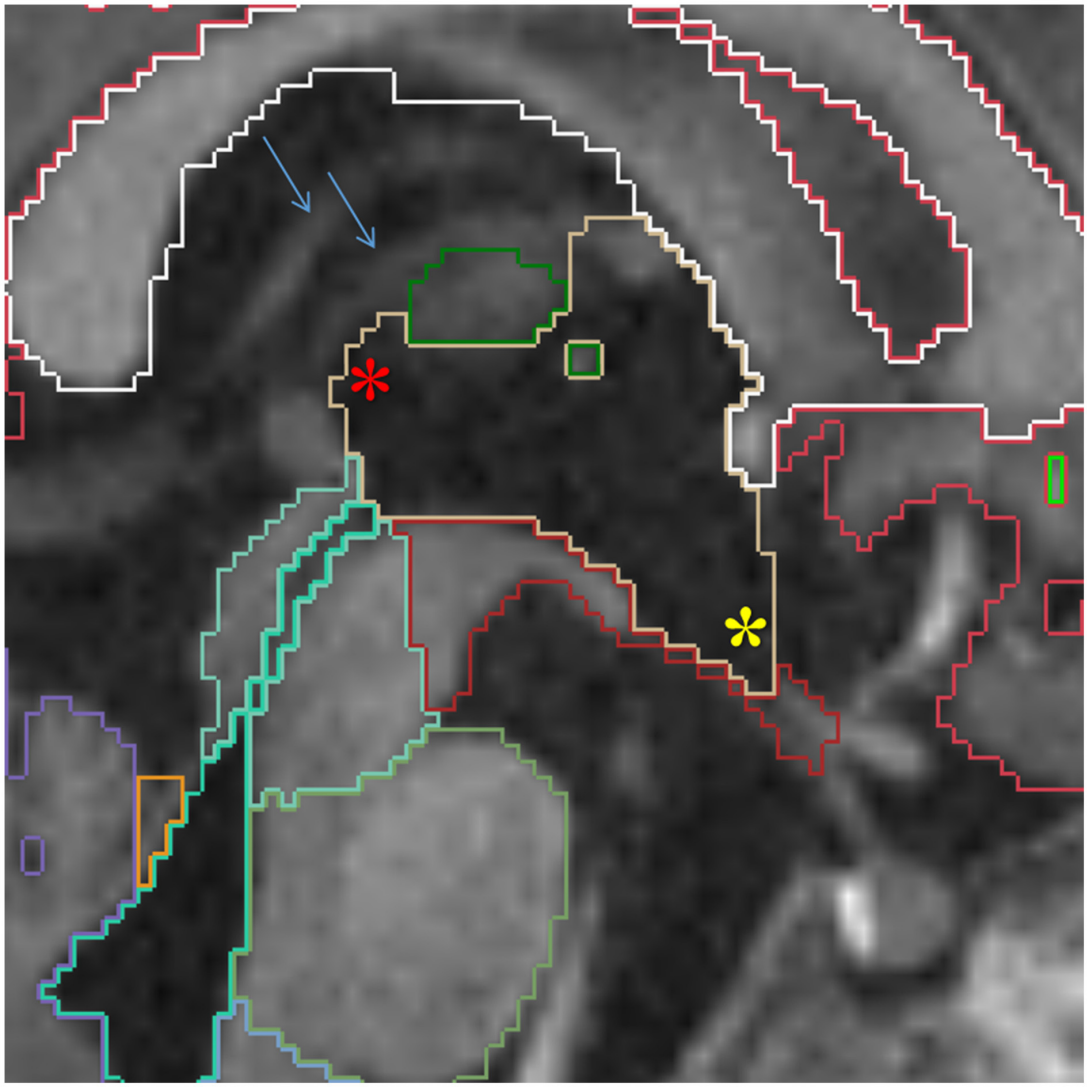
**Sagittal view of the third ventricle (yellow)**. The supra-pineal (red star) and infundibular (yellow star) recesses are shown. The blue arrows point to the internal cerebral veins, which can be used to identify the transverse fissure, indicating the third ventricle should not be prolonged this far posterior. Other labels in this figure: white matter (white), cerebral cortex (red), mesencephalon (turquoise), pons (green) and medulla (baby blue), VDC (dark red), fourth ventricle-cerebral aqueduct (lime green) and vermis (violet).

This central diencephalic CSF-filled cavity provides communication between the lateral ventricles and the fourth ventricle through the intraventricular foramina superiorly and the cerebral aqueduct inferiorly. When feasible in older infants, an effort should be made to delineate the supraoptic, infundibular, pineal, and suprapineal recesses of the third ventricle in sagittal plane. However, given voxel size limitations, most of the times, only the infundibular and suprapineal recesses are identified (Figure 9).

### Cerebral aqueduct and fourth ventricle

**Figure 10:**
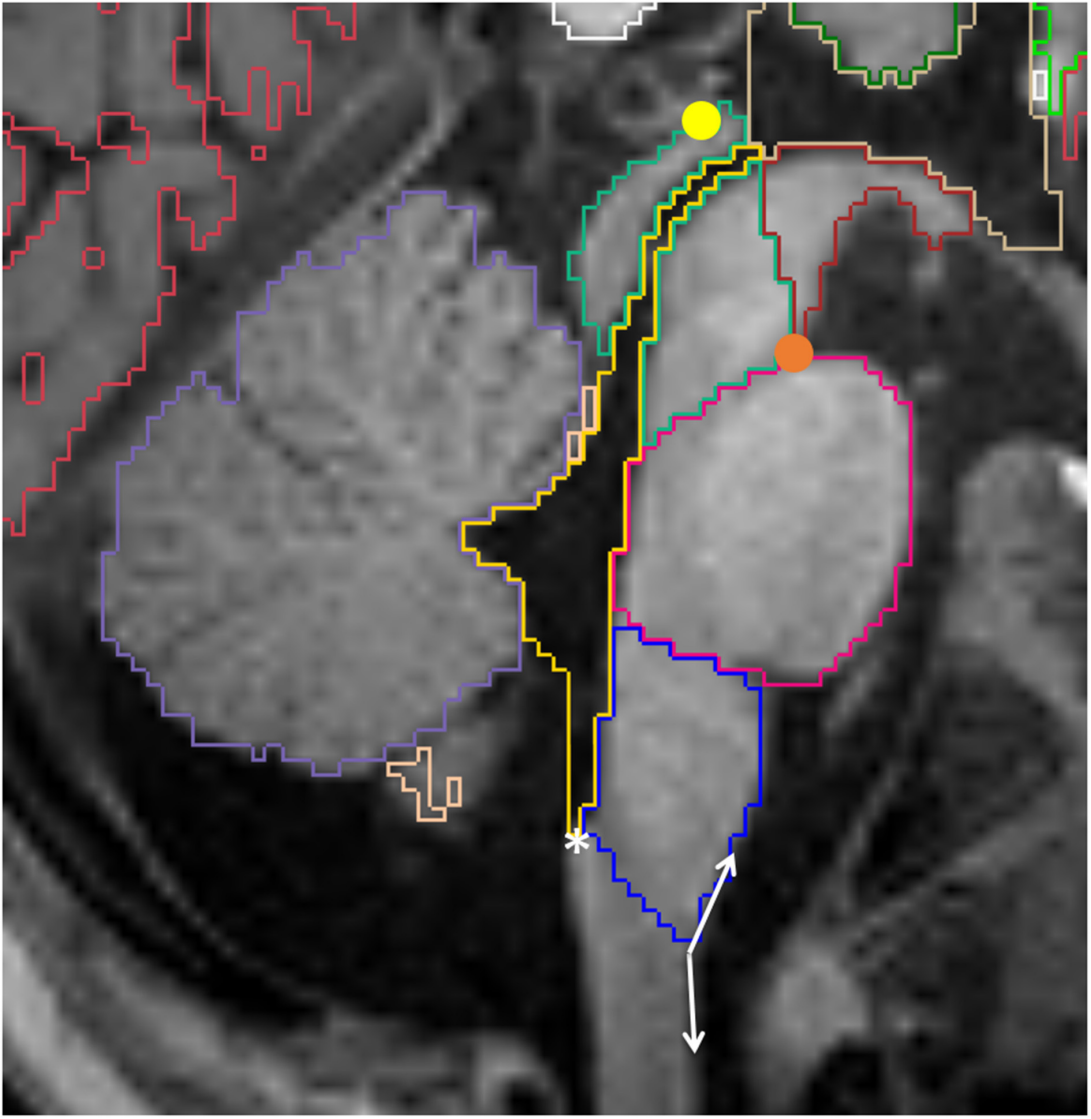
**Sagittal view of the brainstem segmented in a 12 month old**. Mesencephalon (lime green), pons (pink) and medulla (dark blue) are represented, as well as the VDC (dark red), fourth ventricle-cerebral aqueduct (yellow) and vermis (violet). Note the conventional division between VDC and mesencephalon as a line running from the pontomesencephalic sulcus (orange circle) to the posterior commissure (yellow circle). The white asterisk is on the obex, while the double arrow show the obtuse angle that identifies the pyramidal decussasion at its center.

The cerebral aqueduct runs through the posterior part of the mesencephalic tegmentum and provides communication between the third and the fourth ventricles. It receives the same label as the fourth ventricle, which has a diamond shape and lies between the brainstem and the cerebellum (Figure 10). The cerebral aqueduct opens to the extra-cerebral CSF space through the Magendie and Lushka foramina. Due to difficulty determining the inferior limit of the fourth ventricle, the obex should be used as a reference for most inferior point of the fourth ventricle, better appreciated on sagittal images.

### Ventral diencephalon (VDC)

Although we can promptly identify the thalamus on magnetic resonance images, the remaining diencephalic structures are not very well distinguishable from each other and they do not have well-established landmarks to determine individual boundaries, even when considered as a group. For this reason, we implemented a convention for the ventral diencephalic structures, where the thalamus is taken as the superior border posteriorly. On the sagittal images, it is possible to identify theanterior commissure as the most antero-superior limit and the infundibular recess as the most antero-inferior limit. The lateral boundaries should not extend beyond the optic pathways and the posterior-inferior boundary is the mesencephalon.

Because the boundary between the mesencephalon and ventral diencephalon is also obscure, we establish an oblique line running from the most anterior and inferior point of the mesencephalon, at the level of the pontomesencephalic sulcus, to the posterior commisure, with the VDC located above and the mesecephalon below this line (Figure 10). This convention has been described previously by Makris et al. (2004). Although we acknowledge that some mesencephalic structures are incorrectly labeled as VDC, we found this line to be easily and consistently reproducible across different segmenters. Also, since this convention is used by the FreeSurfer package for segmentation of adult brains, future studies would benefit from the possibility of longitudinal analysis.

### Brainstem

**Figure 11:**
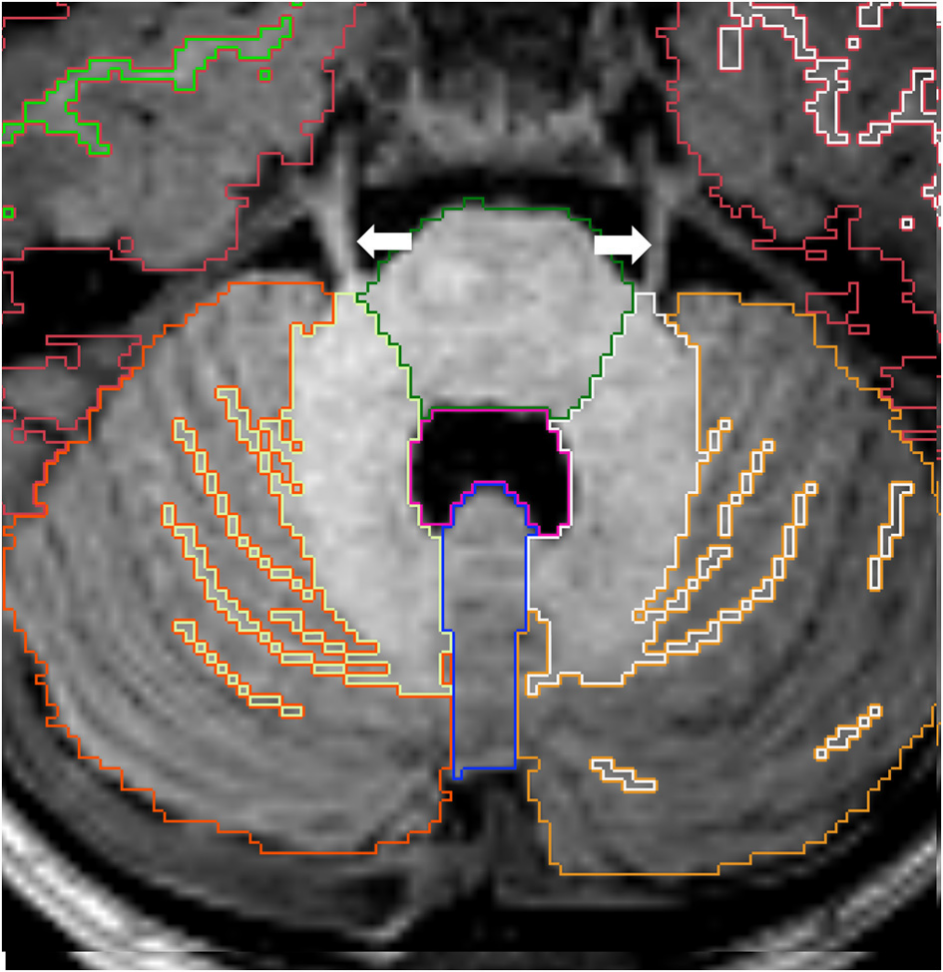
**Axial view of cortex of cerebellar hemispheres (orange), cerebellar white matter (yellow), cerebellar vermis (violet), pons (green) and fourth ventricle (pink)**. The two cerebellar hemispheres are separated by the vermis medially. Note the trigeminal nerves (white arrows) emerging from the brainstem on each side. Other labels: cerebral cortex (red), right cerebral white matter (bright green), left cerebral white matter (white).

The brainstem is subdivided into three regions: mesecephalon, pons, and medulla. The boundaries between the mesencephalon and the VDC are described above. The limits between the mesencephalon and the pons, and between the pons and the medulla, are defined by the pontomesencephalic and bulbopontine sulci, respectively. These are better appreciated on the sagittal images (Figure 10). An oblique line from the obex to the inferior aspect of the pyramidal decussation defines the limit between the medulla and the spinal cord. The obex can be identified as the inferior most portion of the fourth ventricle, where a small step can be identified on the posterior surface of the medulla on sagittal view. The pyramidal decussasion corresponds to the center of the slight obtuse angle on the anterior surface of the cervicomedullary junction (Figure 10). We label the superior and the middle cerebellar peduncles as part of the cerebellum. Increasing the image contrast can help to identify the slight signal difference between the pons (of lower signal intensity) and the middle cerebellar peduncle (of higher signal intensity). Another reference point for this boundary is the emergence of the trigeminal nerves on the surface of the brainstem, with the pons located medially and the middle cerebellar peduncles laterally (Figure 11).

### Cerebellum

The right and left hemispheres of the cerebellum are separated by the vermis, which constitutes the roof of the fourth ventricle (Figure 11). The boundaries between the cerebellar vermis and hemispheres are best visualized in the axial plane.

For subjects in which the cortex-white matter interface is delineated, we also segment the cerebellar cortex-white matter boundary. In these cases, the deep cerebellar gray nuclei and the cerebellar peduncles are included within the cerebellar white matter label due to limited imaging resolution. We also try to include as much peripheral projection of the white matter as possible. However, many times the most peripheral lamellae cannot be labeled as white matter even if visually distinguishable from the cortical gray matter. This limitation is related to voxel size, since one single voxel may include the entire section of the cerebellar folia.

### Cerebral hemispheres

**Figure 12:**
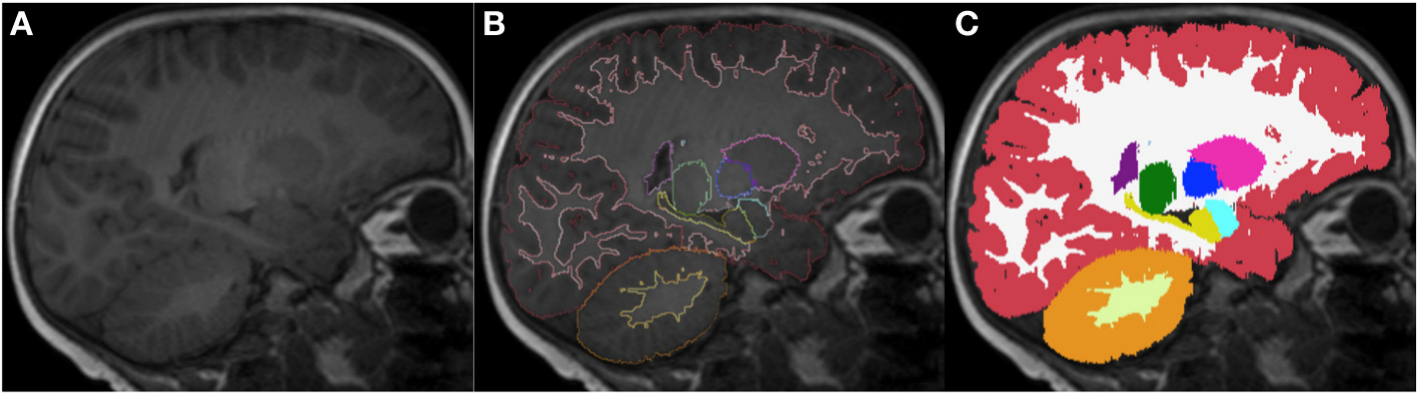
**Sagittal images of an 18-month-old (A) with the respective segmentation, which include cerebral and cerebellar cortex-white matter separation, shown as label outline only (B) and label overlaying the original image (C)**. Labels: cortex (red), left cerebral white matter (white), putamen (pink), pallidum (dark blue), thalamus (green), hippocampus (yellow), amygdala (celeste), cerebellar cortex (orange) and cerebellar white matter (light yellow).

The transverse cerebral fissure, Lateral or Sylvian sulcus, central sulcus, cingulate, superior temporal, calcarine and parietal occipital sulci were delineated in all of our subjects. For the subjects that were 12 months of age or older, we also segmented the cerebral and cerebellar cortex-white matter (Figure 12). At this age, most of the cerebral white matter has an adult-like pattern on T1-weighted images, with the exception of a few areas of late myelination. In the neonatal brain, the white matter is mostly unmyelinated and the free water content is increased relative to the adult, causing the white matter to have lower signal intensity than the gray matter. Therefore, segmentation of cortex-white matter was also performed for neonates, where the contrast between cortex and unmyelinated subcortical white matter could be clearly visualized. After the neonatal period and before 1 year of age, the myelin maturation process causes the cortex-white matter boundaries to become blurred and unreliable for proper segmentation on T1-weighted images.

## Results

**Figure 13:**
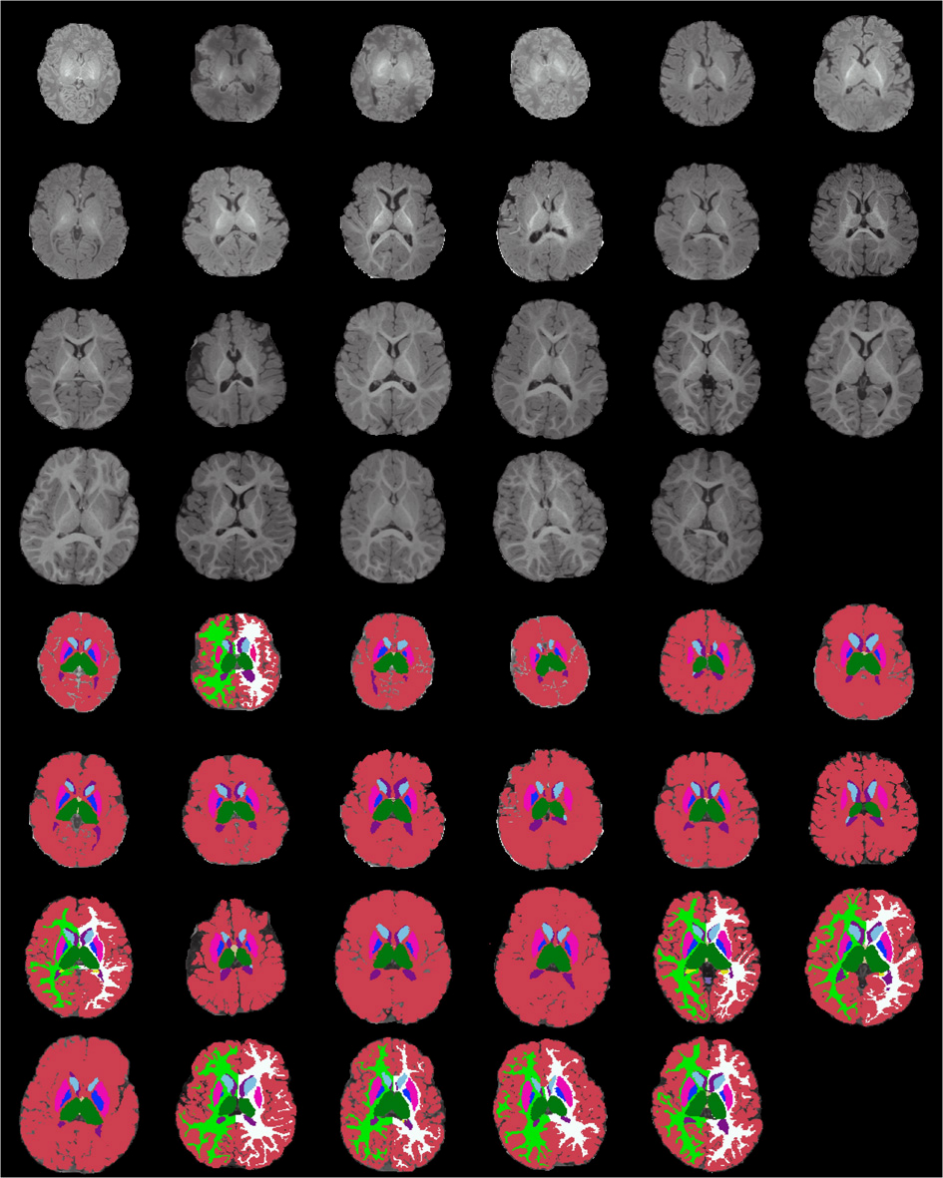
**Age-sorted axial images of all structural acquisitions and their corresponding manual segmentations**. All images are displayed in their native coordinate space, without being spatially registered. Only images with sufficient gray-white contrast have white matter manually labeled.

**Figure 14:**
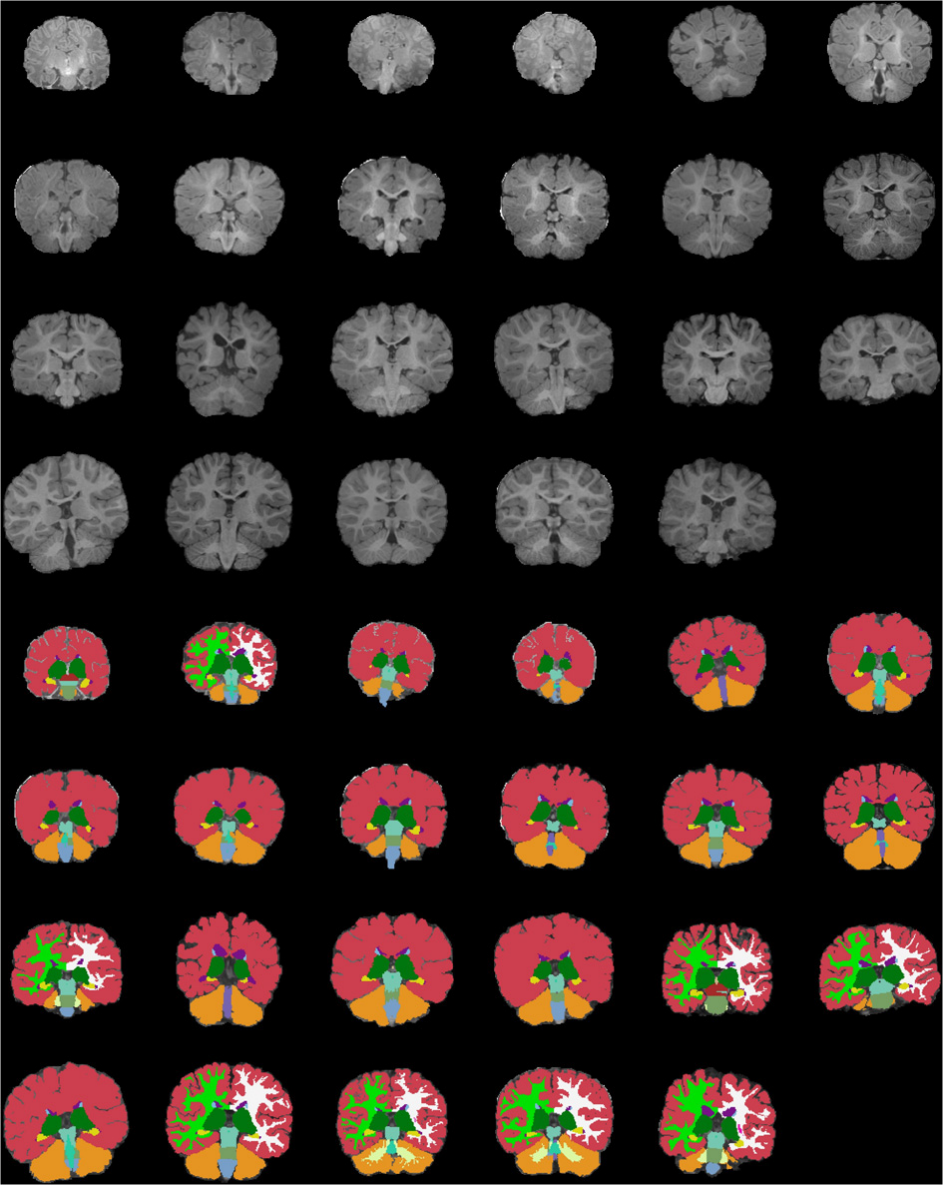
**Age-sorted coronal images of all structural acquisitions and their corresponding manual segmentations**. All images are displayed in their native coordinate space, without being spatially registered. Only images with sufficient gray-white contrast have white matter manually labeled.

**Figure 15:**
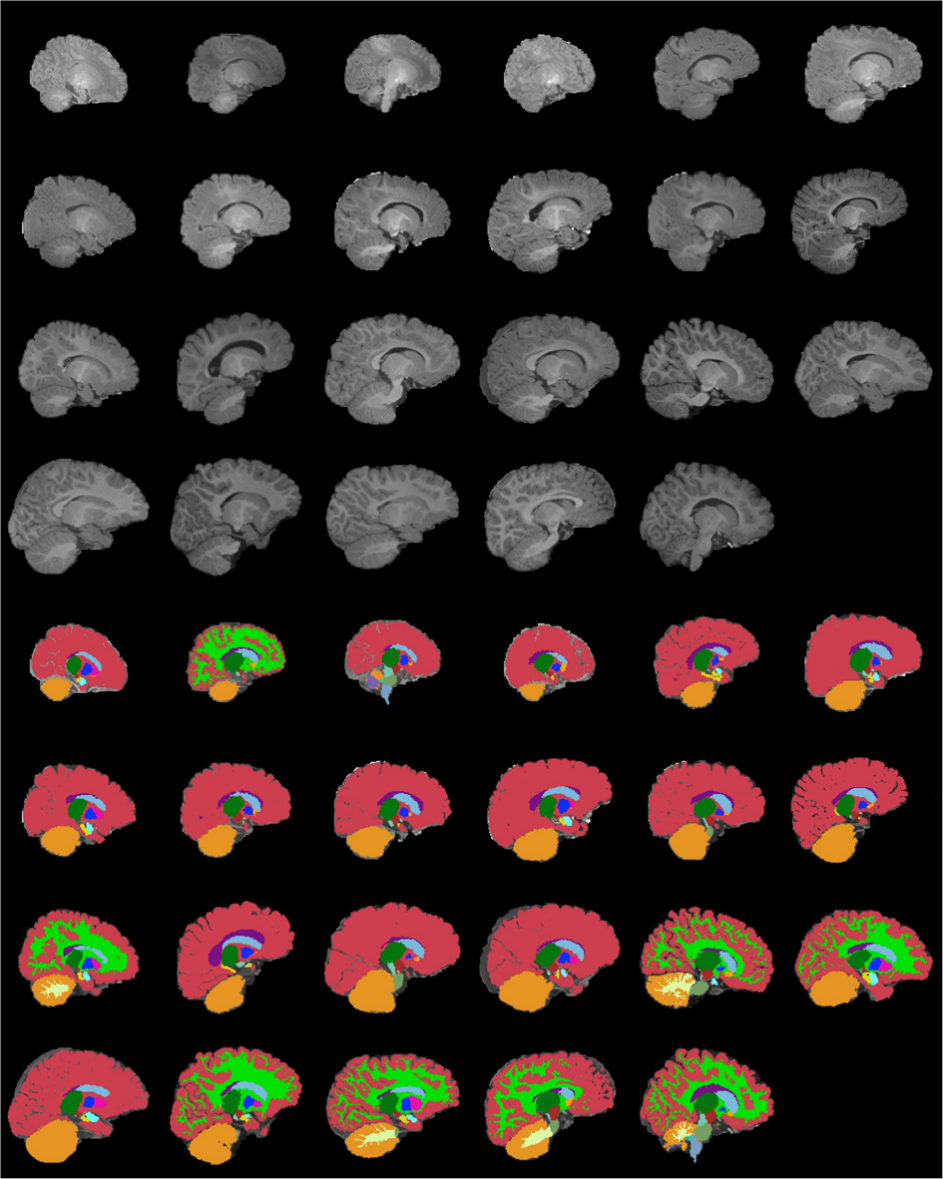
**Age-sorted sagittal images of all structural acquisitions and their corresponding manual segmentations**. All images are displayed in their native coordinate space, without being spatially registered. Only images with sufficient gray-white contrast have white matter manually labeled.

Figures 13–15 display axial, coronal and sagittal images of all our age-sorted structural acquisitions in the training data set and their corresponding manual segmentations. All images are displayed in their native coordinate space. The acquisitions among the different subjects are not spatially registered.

### Inter-rater variability of the segmentations

We have mentioned at the beginning of this document that in order to maintain accuracy and consistency of our segmentations (completed by a set of segmenters), all manual labelings were reviewed, corrected and finalized by the same neuroradiologist (KMR). In this section, we also provide information about inter-rater variability that was computed after repeating the manual segmentation of a subset of the data sets. In fact, we carried out two independent inter-rater variability studies relying on segmenters at two different institutes. One set of studies was carried out at our own institution on a subset of the data set that we have described above and the other one at a collaborating institute with labelers trained in our group using MRI sequences acquired in a comparable set up. In the former scenario a new labeler (trained by the here presented material) has re-segmented 10 labels (L/R Thalamus, L/R Putamen, L/R Pallidum, L/R Hippocampus, L/R Amygdala) on five subjects (of ages 0, 3, 6, 12, and 18 months) and in the latter two labelers independently segmented 23 labels (L/R Lateral Ventricle, L/R Cerebellum Cortex, L/R Thalamus, L/R Caudate, L/R Putamen, L/R Pallidum, 3rd Ventricle, L/R Hippocampus, L/R Amygdala, L/R Accumbens Area, Vermis, Midbrain, Pons, Medulla) on 3 MRI volumes of subjects (of 8, 12, 16 days of age). The mean and standard deviation of the Dice overlap coefficients from these segmentations are displayed on Supplementary Figures 1, 2. In the case of the BCH data set, the worst performance (<60%) is observed in the case of the L Amygdala. For the R Amygdala the repeatability is over 60%. There are four structures for which the performance is between 70 and 80% (L/R Pallidum and L/R Hippocampus), three between 80 and 90% (L/R Putamen and R Thalamus) and one above 90% (L Thalamus). The two highest numbers correspond to the segmentation of the L and R Thalamus. In the case of the collaborator data set, it is the L and R Accumbens that performed worst with the 3rd Ventricle, L Pallidum and L Amygdala also getting low performance percentages. In total, there were 9 labels where the overlap is greater than 80% (L/R Thalamus, L/R Caudate, L/R Putamen, Vermis, Midbrain, Pons), four between 70 and 80% (L/R Lat Ventricle, L/R Hippocampus, R Pallidum, Medulla) and four between 60 and 70% (L Pallidum, 3rd Ventricle, L/R Amygdala).

## Discussion

We present guidelines to consistently label regions of interest in infant brain MRI images, and a training data set of acquisitions that were all segmented according to these principles. Our data set is populated with subjects whose ages are approximately uniformly distributed in the 0 to 2-year range. Although Shi et al. (2011) gathered a significant database with neonates to 2-year old infants, they only have 3 representative time points (neonates, 1 and 2 year old) and their atlas is derived from automated parcellation. We believe that having multiple time points along the age distribution will make it easier to interpret and account for age-related anatomical characteristics. Sanchez et al. (2012) have also built an atlas using subjects of multiple ages (2 weeks, 3, 4.5, 6, 7.5, 9, 12, 15, 18 months, 2, 2.5, 3, 4 years), however, their data is mostly based on 3 mm thick 2D images acquired on a 1.5 Tesla scan, which is not ideal for most volumetric post-processing purposes.

Another positive characteristic of our initiative is the fact that we delineated multiple structures, as opposed to being restricted to GM, WM and CSF. Gousias et al. (2012) described a rich and detailed protocol for infant manual segmentation, but it was implemented only for newborns.

Our analysis was all performed using a single MRI sequence. This was a retrospective study, where we could not optimize sequences for contrast/time or distortion reduction. Moreover, only the acquired MPRAGEs were true volumetric sequences and had correction for motion, with T2- and diffusion-weighted images acquired in a 2D mode and without motion correction. We are aware that the single channel approach can impose some limitations when determining boundaries and impeding the cortex-white matter, as well as myelinated-unmyelinated white matter differentiation. However, we have found that at the level of detail we were segmenting, the co-registration of different MRI sequences was not always helpful for our segmenters, and at times was even misleading due to boundary shifts between the different channels, caused by differential distortions in the different types of acquisitions. The use of a single channel is possibly valuable due to the fact that it might be easier to perform in future studies and more applicable to clinical data, which often cannot be a lengthy and detailed study.

Most of our labels are present in all the subjects, with the exception of the cortex-white matter differentiation, which was obtained only for neonates and after the first year of life. The difficulty in separating those two structures is a consequence of the rapidly evolving white matter contrast in the first year of life, secondary to the myelin maturation process. Neonates have fewer areas of complete myelination evident on the T1-weighted sequence, making it easier to visualize the cortex-white matter junction, when compared to subjects a few months older, who have blurred cortex-white interfaces as myelination evolves. At the age of 1 year, most of the brain has a mature myelination pattern, with the exception of some late myelination areas, such as pre-frontal and anterior temporal subcortical U-fibers. This allows for distinction of the contrast between the cortex and white matter.

Due to limited spatial resolution, sulcal delineation is also a challenge. With a 1 mm resolution, adjacent cortical areas and interposed sulci may share a voxel. Because of this, we only fully delineate major sulci. Although some degree of tertiary gyral maturation occurs after birth (Armstrong et al., 1995), most gyral development occurs during the third trimester of gestational life, with the main sulci having a relatively constant development across subjects (Bendersky et al., 2006). In the future, this may allow for the delineation of particular sulci to be used as landmarks for brain orientation and the alignment of subcortical structures in our population, ultimately aiding in the development of strategies for automated segmentation. As a consequence of the missing fine cortical surface delineation and limited cortex-white matter separation, we did not perform cortical thickness estimation.

In spite of the disadvantage of the cross-sectional nature of our data set, the rich information provided with respect to structures, volume, shape, and location and their relation to neighboring structures, combined with the fact that it was developed in a platform that is FreeSurfer compliant, will allow us to use this data set as the basis for the development of a pediatric automated segmentation package. We expect that the implementation of fully automated segmentation will allow for the processing of larger datasets and that we will eventually be able to delineate finer details in individual structural brain development. The atlas can also be invaluable as a tool for medical and neuroscience teaching and as a template to define spatial localization across different imaging methods and other diagnostic tests, such as EEG and MEG.

In summary, we have presented the description of an exceptional data set of fully manually segmented infant brains, with a representative number of subjects evenly distributed between 0 and 2 years of age and a significant number of delineated labels. This dataset has a wide range of potential uses in medicine and neuroscience.

### Conflict of interest statement

BF has a financial interest in CorticoMetrics, a company whose medical pursuits focus on brain imaging and measurement technologies. BF's interests were reviewed and are managed by Massachusetts General Hospital and Partners HealthCare in accordance with their conflict of interest policies.

## Footnotes

^1^http://surfer.nmr.mgh.harvard.edu/fswiki/FreeviewGuide
