# Supplementary figures and images for: A FreeSurfer-compliant consistent manual segmentation of infant brains spanning the 0–2 year age range
Source: Front Hum Neurosci. 2015 Feb 18;9:21. doi: 10.3389/fnhum.2015.00021 (PMC4332305; doi:10.3389/fnhum.2015.00021)

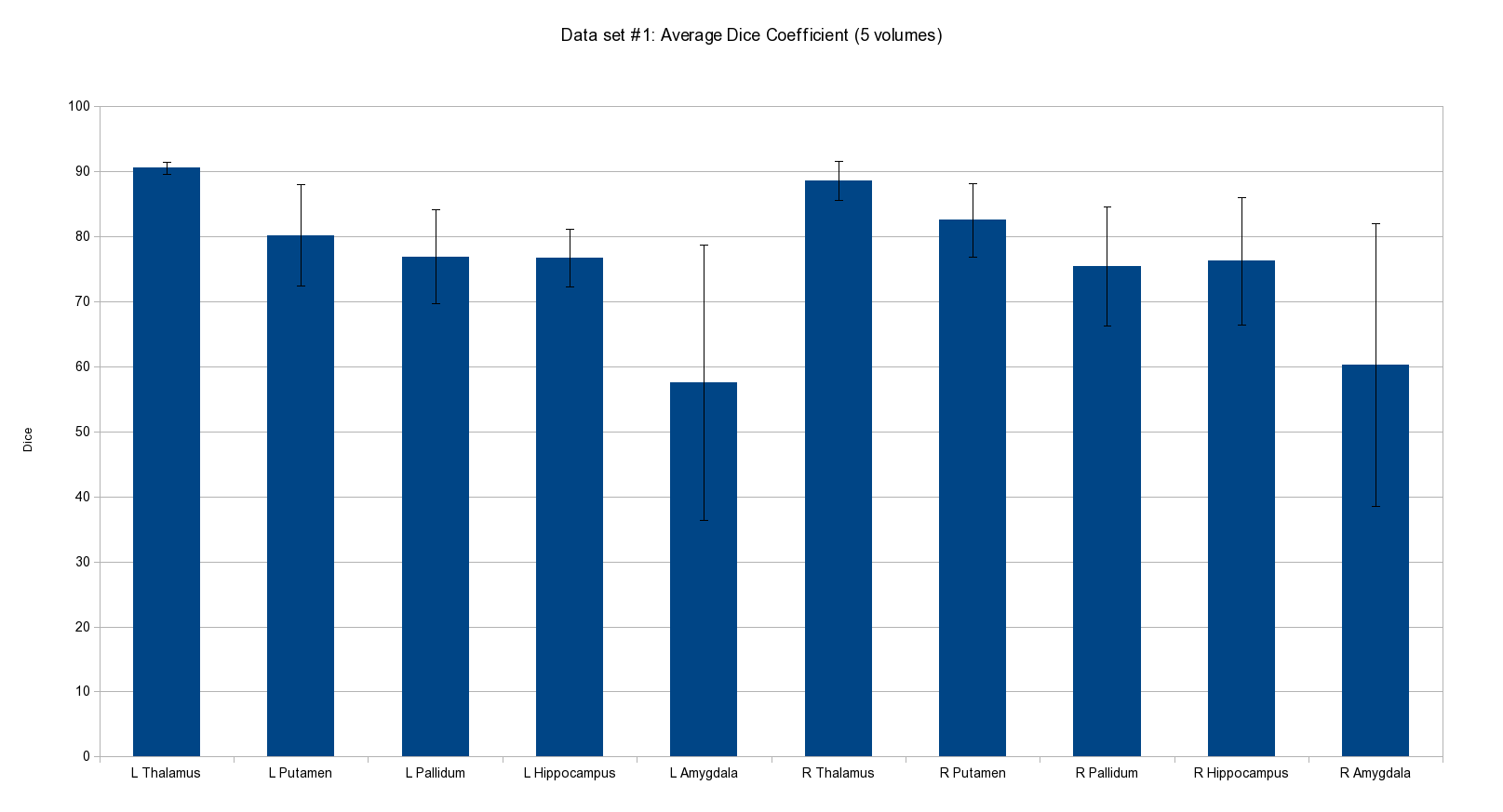

Supplement: Supplementary file 1 [file Image1.TIF]

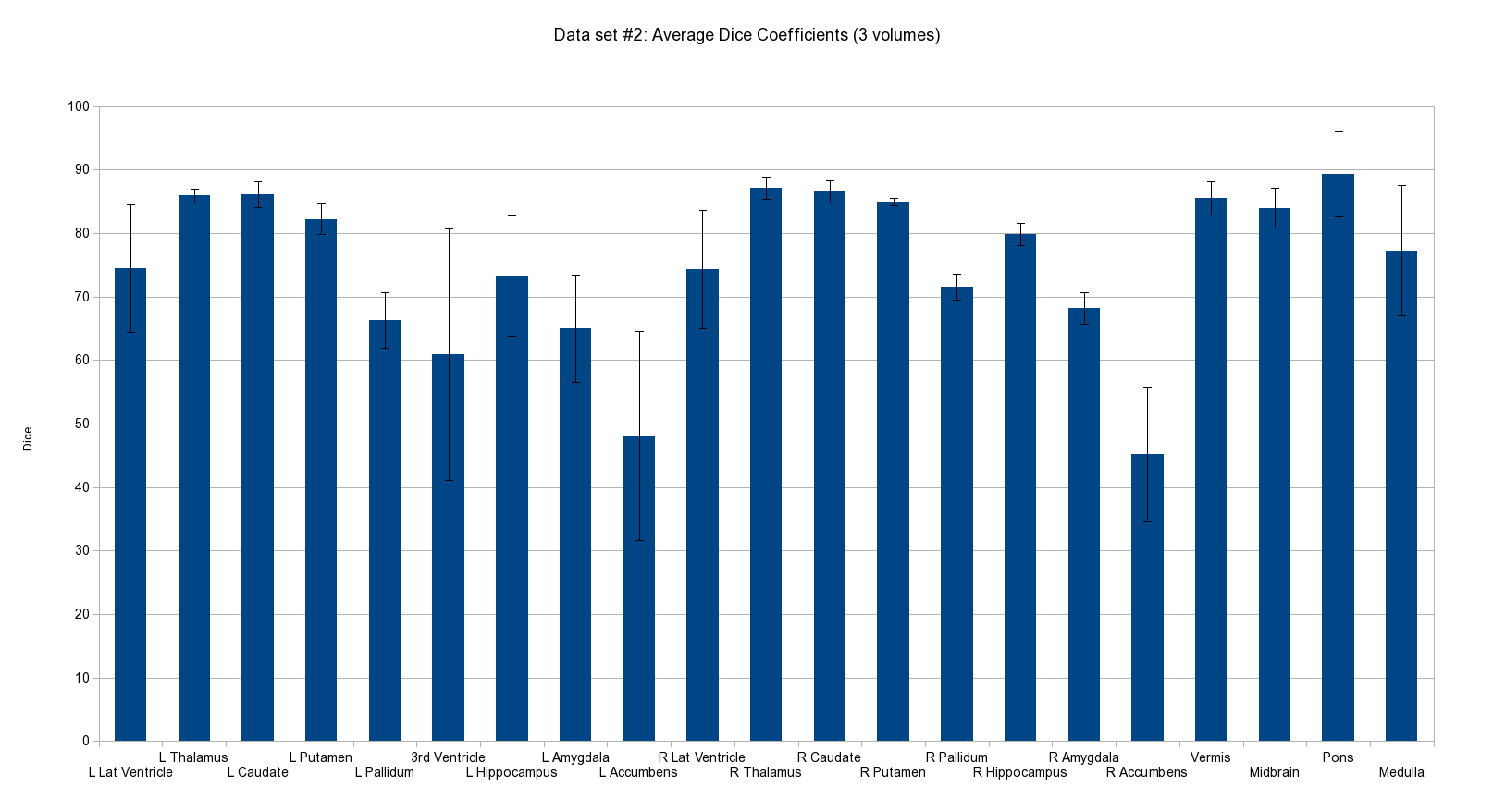

Supplement: Supplementary file 2 [file Image2.TIF]
